# The role of N6-methyladenosine modification in the life cycle and disease pathogenesis of hepatitis B and C viruses

**DOI:** 10.1038/s12276-021-00581-3

**Published:** 2021-03-19

**Authors:** Geon-Woo Kim, Aleem Siddiqui

**Affiliations:** Division of Infectious Diseases, Department of Medicine, University of California, San Diego, La Jolla, CA 92093 USA

**Keywords:** RNA editing, Hepatitis B, Hepatitis C

## Abstract

N6-methyladenosine (m^6^A) is the most prevalent modification of mammalian cellular RNAs. m^6^A methylation is linked to epigenetic regulation of several aspects of gene expression, including RNA stability, splicing, nuclear export, RNA folding, and translational activity. m^6^A modification is reversibly catalyzed by methyltransferases (m^6^A writers) and demethylases (m^6^A erasers), and the dynamics of m^6^A-modified RNA are regulated by m^6^A-binding proteins (m^6^A readers). Recently, several studies have shown that m^6^A methylation sites have been identified in hepatitis B virus (HBV) transcripts and the hepatitis C virus (HCV) RNA genome. Here, we review the role of m^6^A modification in HBV/HCV replication and its contribution to liver disease pathogenesis. A better understanding of the functions of m^6^A methylation in the life cycles of HBV and HCV is required to establish the role of these modifications in liver diseases associated with these viral infections.

## Hepatitis: Role of RNA modification

Further investigations into the role of RNA modifications during hepatitis B and C infections could improve understanding of the hepatitis-associated liver disease. The most common RNA modification found in mammalian cells is N6-methyladenosine (m^6^A), which is linked to multiple cellular processes including gene regulation. This modification has also been identified in viral RNA, prompting Geon-Woo Kim and Aleem Siddiqui at the University of California, San Diego, USA, to review understanding of m^6^A modification during hepatitis virus infections. Depending on which part of the viral RNA is m^6^A-methylated, m^6^A modification induces complex changes in the viral life cycle. Infection with either hepatitis B or C virus also affects m^6^A modifications in host cellular RNA, influencing gene expression and inhibiting immunity. This may influence the development of liver disease and cancer during chronic hepatitis infection.

## Introduction

**Fig. 1:**
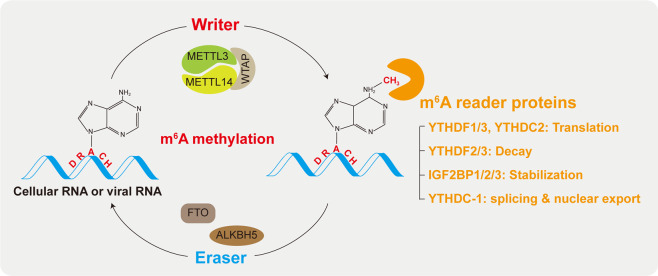
The roles of the cellular m^6^A machinery and m^6^A reader proteins in regulating cellular RNA and viral RNA. m^6^A modification occurs in consensus DRACH motifs of cellular and viral RNAs. This modification is reversibly catalyzed by an m^6^A ‘writer’ or ‘eraser’. The m^6^A ‘writer’ (methyltransferase) complex is composed of METTL3, METTL14, and WTAP, and FTO or ALKBH5 is m^6^A ‘eraser’ (demethylase). The dynamics of m^6^A-modified RNAs are regulated by the m^6^A ‘reader’ proteins, including YTHDF1/2/3, YTHDC1/2, and IGF2BP1/2/3.

Eukaryotic cellular RNAs contain diverse chemical modifications, including N6-methyladenosine (m^6^A), 5-methylcytidine (m^5^C), uridine to pseudouridine (U to Ψ), adenosine to inosine (A to I), and addition to N7-methylguanosine (m^7^G)^1^. Among the diverse RNA modifications, m^6^A methylation, methylation of the adenosine base at the nitrogen 6 position, is the most well-characterized and the most common modification of cellular RNAs^2,3^. This modification has been linked to various biological processes, including innate immune responses, sex determination, stem cell differentiation, circadian clock regulation, meiosis, stress responses, and cancer development^3^. m^6^A methylation was first identified in the 1970s but the technology to map individual-specific m^6^A sites in a given RNA became available only recently^4^. The development of highly sensitive detection methods with high-throughput sequencing revealed the topology of m^6^A in the cellular transcriptome^2,5^. Over 25% of mammalian transcripts contain m^6^A modifications and m^6^A modification occurs within the consensus DRACH/RRACH motif (D = A, G, or U; R = G or A; H = A, C, or U)^2^. Furthermore, this modification is typically enriched near the stop codon and the 3′-untranslated region (UTR)^2^. Similar to DNA methylation, m^6^A methylation is reversibly catalyzed by various methyltransferases and demethylases (Fig. 1). The cellular m^6^A methyltransferase machinery is composed of methyltransferase-like 3 (METTL3), METTL14, and WT1-associated protein (WTAP)^6,7^. Other additional subunits, such as Vir like m^6^A methyltransferase associated (VIRMA), zinc finger CCCH-type containing 13 (ZC3H13), and RNA-binding motif protein 15/15B (RBM15/15B), are also components of the m^6^A machinery^8–11^. WTAP regulates the recruitment of the optimal substrate and nuclear localization of METTL3/14^8^. RBM15/15B interacts with the U-rich RNA regions, ZC3H13 is required for nuclear import of the METTL3/14 complex, and VIRMA is necessary for writing m^6^A in the 3′-UTR^9–11^. Fat mass and obesity-associated protein (FTO) and AlkB homolog 5 (ALKBH5) are m^6^A demethylases that remove m^6^A from cellular RNA (Fig. 1)^12,13^. The dynamics of m^6^A modified RNAs are regulated by m^6^A readers, YT521-B homology (YTH) domain family proteins (YTHDF1/2/3 and YTHDC1/2)^14^. YTHDF3 first recognizes m^6^A-modified RNA and recruits the YTHDF1 or 2 protein^15^. The YTHDF1-YTHDF3 complex induces the translation of m^6^A-methylated mRNA, while the YTHDF2-YTHDF3 complex causes the degradation of its target mRNA degradation^16,17^. Thus, YTHDF3 regulates mRNA degradation and translation by cooperating with YTHDF1 or 2. Because YTHDF2 has no RNase activity, it interacts with the CCR4-NOT (C-C motif chemokine receptor 4 negative on TATA-less) deadenylase complex to promote the degradation of its target RNA^17^. YTHDC1 regulates mRNA nuclear export in cooperation with the major export receptor NXF1, as well as RNA splicing^18,19^. YTHDC2 is the only m^6^A reader protein containing an RNA helicase domain and induces the translation of m^6^A-modified mRNA by interacting with a small ribosomal subunit^20,21^. The helicase activity of YTHDC2 is essential for YTHDC2-mediated mRNA translation, implying that YTHDC2 helps to resolve mRNA secondary structure^22^. Thus, m^6^A-methylated RNAs are epigenetically regulated by diverse m^6^A-binding proteins. However, the mechanism by which the m^6^A site recruits specific m^6^A binding proteins remains to be elucidated.

Several recent reports highlighted the role of m^6^A in the genomes of RNA viruses as well as in the transcripts of DNA viruses^23–32^. m^6^A modification can affect viral life cycles in a complex way. Viral RNAs can be m^6^A methylated; therefore, m^6^A can play an antiviral or proviral role in the viral life cycle through the recruitment of different m^6^A-binding proteins. In addition, m^6^A can indirectly affect viral replication by regulating the expression of specific genes involved in the viral life cycle. A better understanding of the biological functions of m^6^A modification in viruses is important to establish their role in viral pathogenesis and to design innovative prevention measures to affect viral infection. In this review, we will summarize the emerging roles of m^6^A modifications in HBV and HCV infections and discuss their functions and associated mechanisms related to the biological processes of viral infection.

## The role of m^6^A during hepatitis B and C virus infections

### The role of m^6^A in the HBV life cycle

HBV infection leads to chronic hepatitis and carries a risk for the development of hepatocellular carcinoma (HCC)^33,34^. HBV belongs to the *Hepadnaviridae* family and contains a partially double-stranded DNA genome. Although HBV is a DNA virus, it replicates by reverse transcription of an RNA intermediate termed pregenome RNA (pgRNA) to ultimately produce viral genomic DNA in a covalently closed circular conformation termed cccDNA^35^. Initially, pgRNA is reverse transcribed to relaxed circular DNA (rcDNA) in the cytoplasmic core particles, and rcDNA is subsequently converted to cccDNA in the nucleus, where it functions as a template for transcription^34^. Transcription from cccDNA is achieved through the cellular polymerase II machinery to synthesize viral RNAs. Synthesis of HBV transcripts is initiated from different transcription start sites in the HBV genome, but it terminates at a common transcription termination signal^34^. Hence, HBV transcripts have different 5′ termini but share a common 3′ terminal sequence. These HBV transcripts encode the following proteins: surface (HBs), precore or ‘e’ (HBe), and core (HBc) antigen, polymerase, and X (HBx) proteins.

**Fig. 2:**
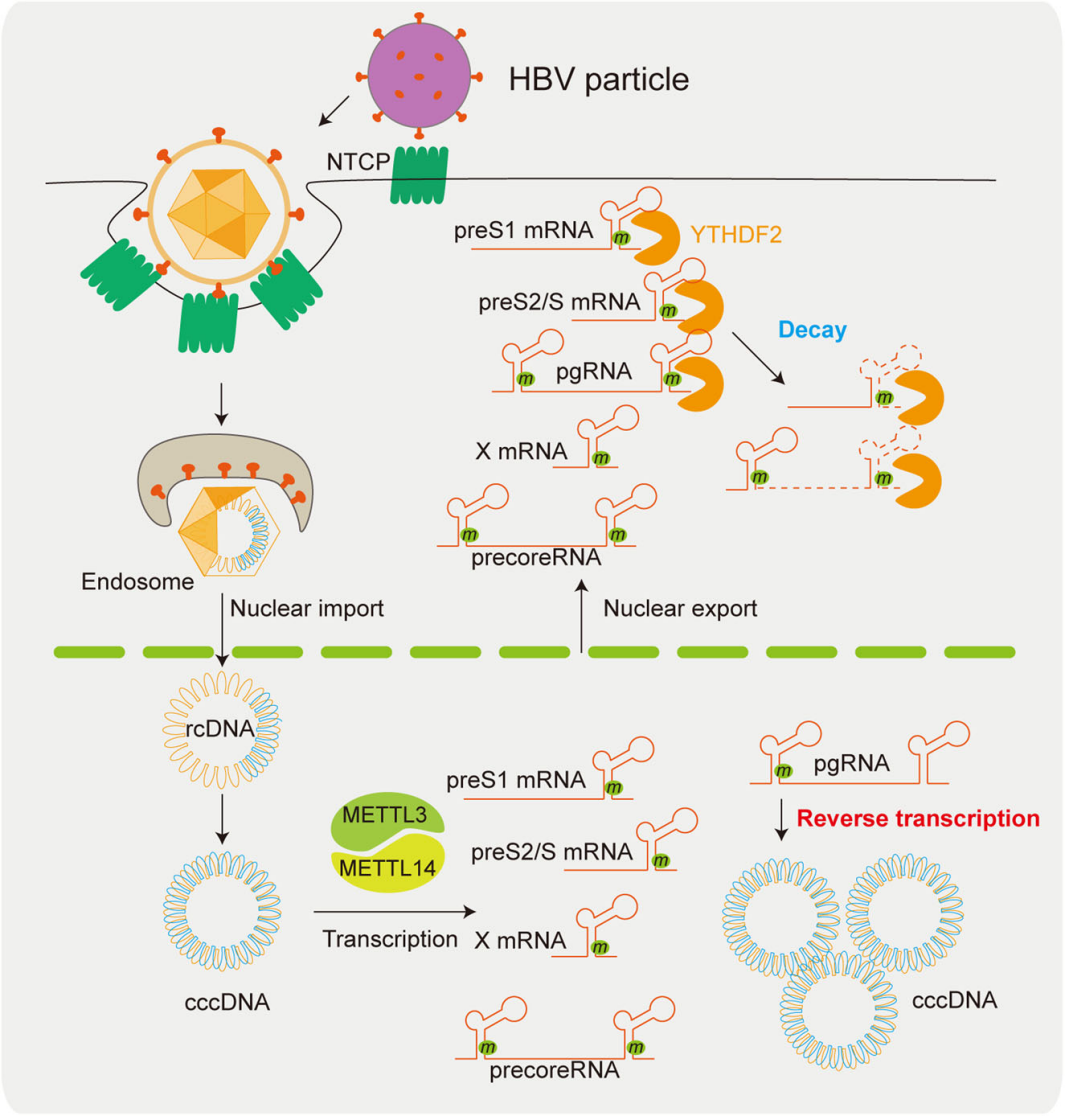
The role of m^6^A modification in differentially regulating the HBV life cycle. HBV transcripts are cotranscriptionally m^6^A-methylated at a consensus DRACH motif in the epsilon stem-loop region. HBV pgRNA contains two such motifs at the 5′ and 3′ termini owing to terminal redundancy, but other viral transcripts contain only one such motif, in the 3′ terminal sequence. m^6^A methylation of the 5′ terminus occurs in the area surrounding the priming site for reverse transcription initiation and induces reverse transcription of HBV DNA from pgRNA, whereas m^6^A at the 3′ terminus in all viral transcripts reduces RNA stability by interacting with YTHDF2.

We first reported that HBV transcripts were m^6^A methylated at an m^6^A consensus motif (A1907) located within the epsilon stem-loop region present in all HBV RNAs^23^. pgRNA of HBV acquires this m^6^A motif at two locations-at the 5′ and 3′ termini due to terminal redundancy, but this motif is presented only once in the 3′ termini of the other subgenomic viral transcripts^34^. Importantly, m^6^A modification of HBV RNAs differentially regulates the viral life cycle depending on its position in the viral RNAs (Fig. 2)^23^. m^6^A modification at the 3′ epsilon stem-loop of HBV RNA transcripts reduces their RNA stability, leading to decreased viral protein expression^23^. The reduction in viral RNA stability resulting from m^6^A is mediated by the recognition of the m^6^A site at the 3′ epsilon stem-loop by YTHDF2 and 3 (m^6^A binding proteins). On the other hand, the m^6^A site located in the 5′ epsilon stem-loop of pgRNA positively regulates reverse transcriptase activity, but the exact mechanism remains to be characterized. Therefore, these results reveal that m^6^A modification in the epsilon stem-loop structure of HBV regulates effects on HBV RNA stability and reverse transcription.

We have recently discovered that HBV utilizes a specific mechanism to guide m^6^A modification on viral RNAs^36^. During HBV infection, HBx interacts with m^6^A methyltransferases, which in turn stimulates their nuclear import and thereby delivers the m^6^A methyltransferases to HBV cccDNA to achieve cotranscriptional m^6^A modification of HBV RNAs. On the other hand, infection with HBx-defective HBV fails to produce m^6^A-modified viral transcripts^36^. In this role, HBx regulates the HBV life cycle by modulating m^6^A modification of viral RNAs. These findings highlight the unique role of HBx in the cotranscriptional RNA modification at the sites of transcription initiation, in addition to its transactivating function affecting the Smc5/6 complex and HBx-DDB-mediated degradation activity^37–39^.

In addition, m^6^A modification plays an important role in interferon (IFN)-mediated inhibition of HBV replication^40^. IFN treatment of HBV-infected cells promotes the reduction of viral replication through the degradation of viral RNAs by the exonuclease activity of the IFN-stimulated gene 20 (ISG20)^41^. ISG20 induced by IFN treatment is recognized by YTHDF2, and YTHDF2 then deliveries ISG20 to the m^6^A-methylated HBV RNAs for their degradation^40^. Mutation of the m^6^A site of HBV RNA abrogates ISG20-mediated viral RNA degradation. This study shows a new function of m^6^A reader proteins in IFN-mediated HBV RNA degradation.

### The role of m^6^A in the HCV life cycle

Hepatitis C virus (HCV) belongs to the *Flaviviridae* family^42^. HCV is a positive-sense single-stranded RNA virus and encodes a polyprotein of ~3010 amino acids that is cleaved by cellular and viral proteases into structural and nonstructural proteins. The viral polymerase has RNA-dependent RNA polymerase activity to replicate viral RNA from a template RNA.

**Fig. 3:**
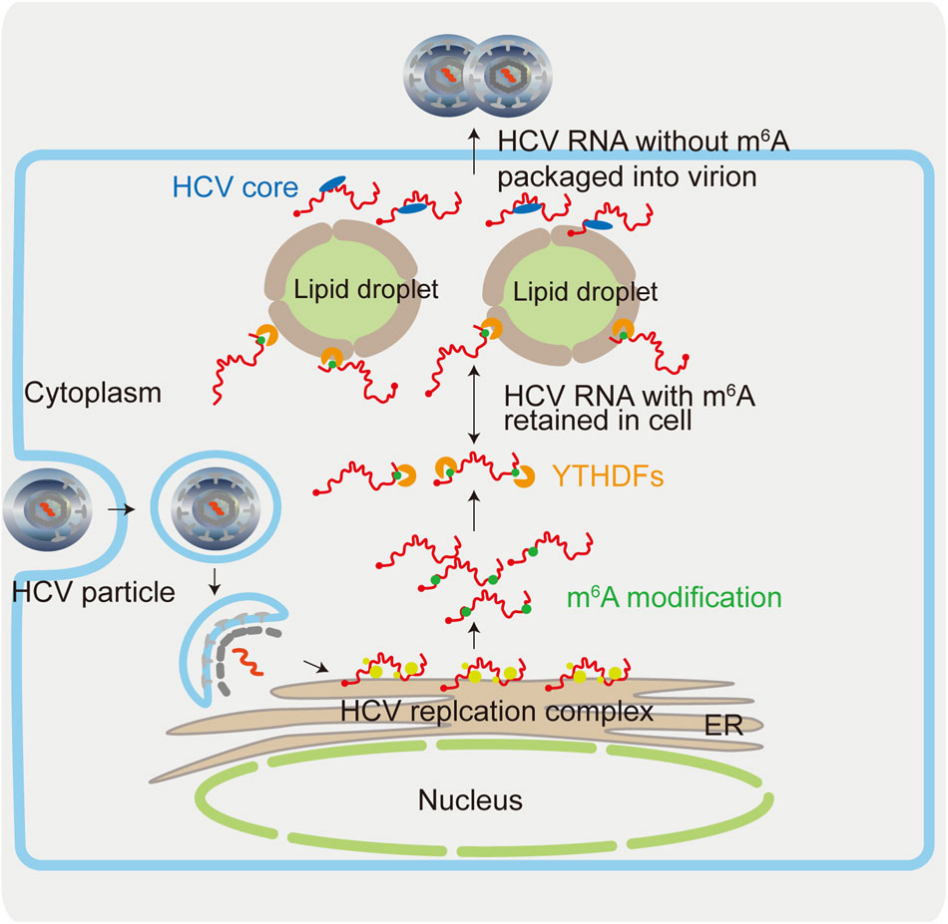
The role of m^6^A modification in regulating the HCV virion packaging. The HCV genome is m^6^A-methylated in several regions (~19 regions), including the HCV E1 region. m^6^A methylations in the HCV E1 region decrease extracellular viral RNA and virion production via recognition by YTHDF proteins. YTHDF proteins sequester the m^6^A-methylated HCV genome to inhibit interaction with the HCV core protein in the lipid droplets.

Horner and colleagues reported that the HCV RNA genome is m^6^A methylated at approximately 19 regions and that all YTHDF proteins broadly interact with the HCV genome^24^. Interestingly, m^6^A modification in the HCV genome decreased extracellular viral RNA levels and viral particle production without affecting viral replication or protein translation. YTHDF1-3 proteins recognized the m^6^A-methylated HCV genome and relocalized HCV RNAs to the lipid droplet fraction to inhibit HCV RNA packaging into virions (Fig. 3)^24^. To elucidate the functional relevance of a specific m^6^A site in the HCV genome, Gokhale et al. mutated m^6^A sites within the HCV E1 coding region. Mutations of these m^6^A sites in the HCV E1 gene increased HCV virion production by abolishing HCV E1 recognition by YTHDF1-3 proteins. These results suggest that m^6^A modifications of the HCV E1 gene regulate viral RNA packaging into virions via interactions with YTHDF1-3 proteins^24^.

Gokhale et al. further analyzed m^6^A motifs in the RNA genomes of other members of the *Flaviviridae* family, including dengue, yellow fever, West Nile, and Zika viruses^24^. Among these viruses, some m^6^A sites were enriched within the NS3 and NS5 regions. Furthermore, HCV, Zika virus, and dengue virus contained similar m^6^A sites in the E1 region. Therefore, these data suggest that potentially conserved m^6^A sites in flaviviruses could regulate the virion maturation process.

### The role of m^6^A in the modulation of host response by HBV and HCV infections

Activation of host pattern recognition receptors (PRRs) by viral infection allows the detection of pathogen-associated molecular patterns and initiates innate immune responses to ultimately eliminate viral infection^43^. PRRs, which detect foreign RNAs, rely on specific molecular signatures and structures to distinguish these RNAs from cellular RNAs and this recognition of foreign RNA is an important cellular surveillance strategy^44^. Interestingly, the ability to use m^6^A to distinguish self from non-self RNAs has been recently highlighted during HBV and HCV infection based on the finding that m^6^A suppresses recognition by retinoic acid-inducible gene I (RIG-I)-like PRRs^45^. The 5′ epsilon structure of HBV pgRNA and the 3′-end poly(U/UC) region of HCV are high-affinity RIG-I ligands^46,47^. m^6^A modifications at the 5′ epsilon stem-loop of HBV pgRNA and the adenosine nucleotide at position 8766 of HCV reduced the sensing activity of RIG-I, while abrogation of these m^6^A sites in HBV and HCV enhanced RIG-I signaling^45^. YTHDF2 interacted with m^6^A sites within RIG-I ligand regions of the HBV and HCV RNAs and inhibited RIG-I signaling. Thus, YTHDF2 may inhibit the sensing of m^6^A-modified viral RNAs by RIG-I by sequestering these RNAs from RIG-I. A similar role of m^6^A in preventing the sensing of viral RNAs by PRRs was also studied in human metapneumovirus (HMPV)^48^. The genome and antigenome of HMPV were m^6^A-methylated and m^6^A modification of the HMPV genomes suppressed RIG-I sensing and subsequent IFN production. In contrast, deficient m^6^A modification in the HMPV genomes increased the recognition by RIG-I, leading to enhanced IFN synthesis. Together, these studies indicate that m^6^A modification of viral RNAs contributes to inhibiting RIG-I sensing through its sequestration by m^6^A binding proteins.

In addition to regulating the host immune response, viral infection can regulate host gene expression and cellular processes to optimize long-term survival^49–52^. As m^6^A methylation can regulate many cellular pathways, including stress responses and cancer development, its role in viral infection-related cellular gene expression is an important aspect of virus–host interactions^3^. Indeed, several studies have shown that diverse viral infections modulate the m^6^A profile within the host transcriptome^53–56^. We recently analyzed changes in the m^6^A profile of cellular RNAs during HBV infection^53^. Among the host genes whose m^6^A status was dramatically altered by HBV infection was the phosphatase and tensin homolog (PTEN) transcript, which exhibited enhanced levels of m^6^A methylation during HBV infection. Importantly, increased m^6^A modification of PTEN mRNA by HBV decreased its stability, affecting its protein expression. PTEN is a phosphatase of both proteins and lipids that functions as a metabolic regulator as well as a tumor suppressor^57,58^. Chronic HBV infection causes HCC via diverse pathways, including inflammation and oxidative stress pathways^59^. Thus, the decreased PTEN expression by HBV partially explains its role in HBV-associated hepatocarcinogenesis. In addition to its role as a tumor suppressor, PTEN plays an important role in the innate immune response during viral infections^60^. PTEN promotes IRF-3 nuclear translocation to activate the IFN signaling pathway by inducing dephosphorylation at the Ser96 residue of IRF-3. Based on these findings, HBV inhibits the host immune response through upregulation of m^6^A modification of PTEN^53^. Interestingly, the HBx protein cotranscriptionally regulates m^6^A modification of cellular RNA, including that of PTEN^36^. HBx promoted the recruitment of m^6^A methyltransferases (METTL3/14) to the PTEN chromosomal locus to add m^6^A to PTEN transcripts. In addition to its role as a viral protein, the HBx protein is widely acknowledged to be indirectly involved in the development of HCC and viral immune evasion^61,62^. These studies highlight the unique role of the HBx protein in regulating virus/host gene expression, immune responses, and HBV-associated hepatocarcinogenesis by modulating m^6^A modification of cellular RNAs.

HCV infection also regulates host gene expression by modulating m^6^A modification of cellular mRNAs^54^. HCV infection increased the m^6^A level of cellular RIOK3 mRNA, promoting its translation^54^. RIOK3 is a serine/threonine kinase that may be involved in antiviral signaling^63^. Importantly, viral activation of the innate immune response contributed to the increased m^6^A levels of RIOK3, and the increase in RIOK3 expression by m^6^A promoted the production of IFN, leading to inhibition of HCV replication. In addition, the m^6^A level of CIRBP, a stress-induced RNA-binding protein, was changed during HCV infection, although this transcript lost m^6^A modification^54,64^. In the case of CIRBP, m^6^A deficiency promoted alternative splicing to its shorter isoform. Interestingly, endoplasmic stress induced by viral infection promoted the loss of m^6^A in CIRBP, and the expression of the short isoform of CIRBP positively regulated HCV replication^54,65^. The precise mechanisms by which HCV infection changes the m^6^A status of individual transcripts are not clear, but these data suggest that activation of host cell pathways during infection may affect the m^6^A status of individual cellular RNAs.

## Conclusion and future perspectives

New roles of m^6^A in epigenetically regulating cellular RNAs and viral RNAs are constantly emerging. Recently, reports have demonstrated that the genomes of several RNA viruses, as well as the RNA transcripts of DNA viruses, are modified by m^6^A methylation and that this modification of viral transcripts regulates various aspects of the viral life cycle and the development of pathogenesis^23–31^. In this review, we discussed the recently identified functions of m^6^A modification during HBV and HCV infections. m^6^A modification regulates the HBV and HCV life cycles in a complex way because it can differentially affect both viral and host RNAs depending on their location in the genome. Eventually, the regulation of HBV and HCV infections by m^6^A affects the development of liver disease, suggesting that m^6^A modification plays previously undefined roles in regulating the hepatitis B and C virus life cycles.

Generally, histone H3 trimethylation at lysine 36 (H3K36me3) is bound directly by the cellular m^6^A machinery, which in turn promotes the binding of the m^6^A machinery to adjacent RNA polymerase II molecules, thereby transporting the m^6^A machinery to the transcribed nascent RNA to add m^6^A cotranscriptionally^66^. Importantly, m^6^A methyltransferases are present in the cytoplasm as well as the nucleus^67^. Because HCV replication occurs in the cytoplasmic fraction^42^, it is conceivable that m^6^A methylation of the HCV genome may be accomplished by the cytoplasmic methyltransferases. However, the functional roles of cytoplasmic methyltransferases in mammalian cells are not yet clear. To gain a broad understanding of the mechanism by which the m^6^A machinery and its bound cellular proteins regulate viral infection, future research must address the roles of both the cytoplasmic and nuclear m^6^A machinery in the regulation of viral infection and cellular pathways. Furthermore, an understanding of how and whether viral infections regulate the function of the cellular m^6^A machinery and the m^6^A profiles of host RNAs are needed to enhance our understanding of the role of m^6^A in virus–host interactions. This understanding may offer novel avenues for possible m^6^A-based therapeutic interventions to promote viral genome clearance from infected cells. In addition, m^6^A reader proteins are known to interact with many RNA-binding proteins, suggesting that these interactions can affect viral replication and translation^17,21^. Hence, the interactome of the m^6^A binding proteins during viral infection needs to be identified, which may reveal the unique roles of the RNA-binding protein network that affects the viral life cycle.

Recent studies have highlighted the distinct role of m^6^A methylation in differentiating self RNAs from non-self RNAs based on the findings that m^6^A modification reduces recognition by Toll-like receptor 3 (TLR3), TLR7, and RIG-I^68–70^. In this respect, m^6^A methylation may allow self RNAs to be distinguished from non-self RNAs to evade recognition by cellular RNA sensor proteins, which trigger the immune response. In addition to m^6^A modification, several other chemical modifications, including 5-methylcytosine (m^5^C), uridine to pseudouridine (U to Ψ) editing, and adenosine to inosine (A to I) editing, occur in viral transcripts, and the functions of these modifications are being characterized^70–73^. These modifications can also be used by viruses to mimic self RNA and disrupt the host immune response. This interesting issue is currently under further investigation.
